# Orthopedic and Trauma Physicians’ Ability to Identify Selected SUT Reimbursement Amounts in Türkiye: A Cross-Sectional Survey

**DOI:** 10.3390/healthcare14193284

**Published:** 2026-10-02

**Authors:** Ahmet Yiğitbay, Serhat Yıldırım, Nezih Ziroğlu

**Affiliations:** 1 Department of Orthopedics and Traumatology, İzmir Tepecik Training and Research Hospital, University of Health Sciences, 35170 İzmir, Turkey; ahmetyigitbay@gmail.com (A.Y.); serhat9712@gmail.com (S.Y.); 2 Department of Orthopedics and Traumatology, Acibadem University Atakent Hospital, 34303 Istanbul, Turkey; 3 Department of Orthopedic Prosthetics and Orthotics, Vocational School of Health Services, Acibadem Mehmet Ali Aydinlar University, 34638 Istanbul, Turkey

**Keywords:** reimbursement tariffs, SUT, orthopedics, traumatology, physician knowledge, hypothetical clinical scenarios, Türkiye

## Abstract

**Background:** The Turkish Social Security Institution Health Implementation Communiqué (SUT) specifies administrative reimbursement tariffs that are distinct from actual healthcare delivery costs. Physicians’ familiarity with these tariffs can be examined across different areas of orthopedic practice. **Objective:** To assess orthopedic and trauma physicians’ ability to identify selected SUT reimbursement amounts and to describe their responses to hypothetical clinical scenarios. **Methods:** This cross-sectional online survey included 102 orthopedic and trauma physicians practicing in Türkiye, recruited voluntarily through a national professional society e-mail group. Analyses included demographic and professional information, previous healthcare-cost education, 18 single-response multiple-choice tariff questions, and three hypothetical clinical scenarios. Each correct tariff response received one point, yielding an unweighted total of 0–18. Questions had two to four response options with nonuniform monetary intervals; some options were individual amounts. Scenario responses were treated as descriptive rather than measures of individual decision change. **Results:** The mean tariff-response score was 6.02 ± 2.38, with a median of 6 (IQR, 4–8). Overall, 614 of 1836 responses were correct (33.4%). Assuming one correct option per question and uniform random selection, the theoretical expected accuracy was 31.5%. Accuracy ranged from 22.1% for outpatient procedures to 52.9% for implants, although differences in response formats limit comparisons between categories. Six participants (5.9%) reported previous healthcare-cost education. Non-equivalent scenario response formats and internally inconsistent response combinations prevented valid assessment of individual decision changes. **Conclusions:** Participants selected the reference-matching response for approximately one-third of the tariff questions. Interpretation is limited by heterogeneous response options, the possibility of correct answers through guessing, and the voluntary, nonrepresentative sample. The findings concern selected administrative reimbursement amounts and do not measure actual healthcare costs or comprehensive health-economic knowledge. The hypothetical scenario responses do not establish effects of tariff information on clinical behavior, appropriateness, utilization, expenditure, or patient outcomes.

## 1. Introduction

Rising healthcare expenditure has drawn increasing attention to the economic implications of diagnostic and therapeutic decisions. Previous studies have documented inaccuracies in physicians’ estimates of the monetary values associated with the tests and interventions they use, including among experienced clinicians [1,2].

This issue is relevant to orthopedics and traumatology, where diagnostic imaging, outpatient procedures, surgical interventions, and implant selection contribute to healthcare resource use. Sathiyakumar et al. examined expenditure associated with orthopedic practice, including diagnostic imaging [3]. Okike et al. reported limited knowledge of implant acquisition costs among orthopedic surgeons and residents [4]. Studies of physician trainees and orthopedic residents have similarly documented limited familiarity with the charges or monetary amounts associated with commonly requested imaging examinations [5,6]. In Türkiye, Sarbay et al. found limited accuracy among emergency medicine residents in estimating the monetary values assigned to commonly ordered laboratory and imaging tests [7].

Interpreting these findings requires a clear distinction between monetary concepts. Provider costs, hospital expenditures, implant acquisition prices, patient payments, charges, and reimbursement amounts are not interchangeable. In Türkiye, the Social Security Institution Health Implementation Communiqué (Sağlık Uygulama Tebliği; SUT) specifies reimbursement rules and administrative tariffs for covered healthcare services and medical devices [8]. These tariffs represent amounts recognized for reimbursement within the national social insurance system, rather than the actual costs of delivering care. The present study therefore focuses on selected administrative reimbursement amounts.

Similar distinctions apply across healthcare systems. In the United States, the Medicare Physician Fee Schedule uses relative value units for physician work, practice expense, and malpractice expense to determine geographically adjusted payment amounts [9]. In England, the NHS Payment Scheme sets out rules and payment arrangements for NHS-funded secondary healthcare [10]. Although these systems differ from the Turkish SUT framework, they illustrate the importance of identifying the specific monetary concept assessed when interpreting or comparing studies.

The literature spans several areas of clinical practice and examines different monetary concepts, including charges, acquisition prices, and reimbursement amounts. Assessing responses across imaging examinations, outpatient procedures, surgical interventions, and implants within a single reimbursement framework may help characterize physicians’ familiarity with selected administrative tariffs. Such an assessment remains dependent on the items and response options presented and does not constitute a comprehensive measure of health-economic knowledge.

The primary aim of this study was to assess orthopedic and trauma physicians’ ability to identify selected SUT reimbursement amounts for commonly used imaging examinations, outpatient procedures, surgical interventions, and orthopedic implants in Türkiye. The secondary aim was to describe responses to three hypothetical clinical scenarios, including stated preferences under hypothetical disclosure of SUT prices. Scenario responses were treated as descriptive and hypothesis-generating; they were not used to assess changes in actual clinical behavior or effects on healthcare utilization, expenditure, or patient outcomes.

## 2. Materials and Methods

### 2.1. Study Design and Timeline

This cross-sectional online survey was conducted among orthopedic and trauma physicians practicing in Türkiye. Ethical approval was obtained from the Non-Interventional Clinical Research Ethics Committee of the University of Health Sciences İzmir Tepecik Training and Research Hospital on 4 June 2026 (Decision No. 2026/05-29).

The questionnaire was made available online on 18 June 2026 and closed to further responses on 13 July 2026. Neither the closing date nor a target sample size was prespecified. Reaching 100 respondents prompted the decision to close recruitment. This decision was based solely on respondent numbers and was not influenced by response accuracy, subgroup comparisons, or other analytical results. At closure, 102 complete responses submitted between 19 June and 13 July 2026 had been received, all of which were included in the analyses.

### 2.2. Participants and Recruitment

Eligible participants were orthopedic and trauma residents, specialists, and academic physicians actively practicing in Türkiye during the study period. Physicians from other specialties, medical students, retired physicians no longer in active practice, and respondents who submitted incomplete questionnaires were excluded.

The survey invitation was distributed through the e-mail group of the Turkish Society of Orthopedics and Traumatology (TOTBİD). Participation was voluntary, and no random sampling, stratification, regional quotas, or individual participant-selection procedure was used.

Participation was anonymous. The number and characteristics of invitation recipients who did not respond were unavailable; therefore, a response rate could not be calculated and nonresponse bias could not be assessed. Participants’ geographic locations were not collected, and regional coverage could not be verified. The invitation was nationally disseminated, but the sample was not considered nationally representative.

### 2.3. Sample Size

No a priori sample-size calculation was performed. The study used an open, voluntary recruitment approach, and the analytical sample comprised 102 complete responses. The sample size and small numbers in some subgroups limited the precision of subgroup comparisons and multivariable estimates. Nonsignificant findings were not interpreted as evidence of equivalence or absence of an association.

### 2.4. Survey Development and Administration

The investigators developed the questionnaire specifically for this study following a review of the literature on physicians’ knowledge of imaging charges, procedure reimbursement, healthcare-related monetary values, and implant prices. The questionnaire did not undergo formal pilot testing, external expert-panel review, or psychometric validation before administration. It was designed to assess participants’ ability to identify selected SUT reimbursement amounts and to describe their responses to hypothetical clinical scenarios, rather than to provide a validated measure of comprehensive health-economic knowledge.

The questionnaire was administered using Google Forms. Before participation, respondents were informed of the study purpose, the voluntary and anonymous nature of participation, and their right to discontinue the questionnaire. Submission of a completed questionnaire was considered to indicate informed consent. No identifying information was requested. The analytical dataset contained no missing values for the variables included in the analyses.

### 2.5. Variables and Questionnaire Components

The analyses used demographic and professional information, responses to 18 multiple-choice questions concerning SUT reimbursement amounts, and responses to three hypothetical clinical scenarios.

Demographic and professional variables included age group, sex, professional title, institution type, years of experience in orthopedics and traumatology, average number of outpatient clinic days per month, average number of surgical procedures per month, and previous education concerning healthcare costs. Residents were instructed to include their residency training period when reporting professional experience.

The reimbursement section comprised 18 single-response multiple-choice questions: three concerning plain radiography, three computed tomography, two magnetic resonance imaging, four outpatient procedures, three surgical procedures, and three orthopedic implants. Participants selected one monetary amount or interval for each item. One item had two response options, 11 had three, and six had four. Monetary interval widths varied both within and across items, and some options consisted of individual amounts.

The original Turkish questionnaire used the term “cost,” while its instructions explicitly directed participants to base their estimates on amounts specified under the Social Security Institution Health Implementation Communiqué (Sağlık Uygulama Tebliği; SUT). The original terminology is preserved in the supplementary questionnaire and its translation. The analyzed responses are interpreted as familiarity with selected administrative reimbursement amounts rather than actual healthcare delivery costs.

Reference amounts were obtained through the Ministry of Health’s Price Tariffs webpage [8]. These amounts were checked on 18 June 2026. The same reference-based scoring key was applied to all 102 participants.

The three hypothetical clinical scenarios concerned knee pain, suspected occult hip fracture, and ankle trauma. Participants answered questions about their preferred clinical approaches and conditional questions asking whether their decisions would change if SUT prices were shown. These questions used non-equivalent response formats and did not constitute a standardized before-and-after assessment.

### 2.6. Outcome Measures

The primary outcome was the total number of reference-matching responses across the 18 reimbursement questions. Each matching response received one point and each nonmatching response received zero points, yielding an unweighted total score from 0 to 18.

The total score was used as a descriptive count of correct responses, with each item contributing equally. Equal weighting does not imply equivalent item difficulty or measurement properties. The score was not adjusted for guessing or differences in monetary interval width.

Item-level accuracy was calculated as the number of correct responses divided by 102. Category-level accuracy was calculated as the number of correct responses within a category divided by the number of participants multiplied by the number of items in that category. Overall accuracy was calculated using all 1836 item responses.

Assuming one correct option per item and uniform random selection among the available options, the probability of a correct response was 50% for the two-option item, one-third for each three-option item, and 25% for each four-option item. The theoretical expected total score was therefore 0.5 + 11 × (1/3) + 6 × (1/4) = 5.67 out of 18, corresponding to 31.5% accuracy. This value provides a theoretical benchmark and does not establish whether participants guessed. Differences in response formats and interval widths were considered when interpreting comparisons between items and categories.

Additional outcomes included differences in total scores across professional and demographic groups and the distribution of responses to each hypothetical scenario question. No participant-level decision-change score or combined indicator of decision change was calculated.

### 2.7. Statistical Analysis

Categorical variables were summarized as frequencies and percentages. The total tariff-response score was reported as the mean and standard deviation, median and interquartile range (IQR), and observed range. Quartiles were calculated using linear interpolation. All analyses included 102 participants.

For each reimbursement item, accuracy was calculated as the proportion of participants selecting the reference-matching response. Item-level 95% confidence intervals were calculated using the Wilson method. Overall and category-level accuracy were calculated from each participant’s proportion of correct responses across the relevant items. Their 95% confidence intervals were calculated using the Student t distribution with 101 degrees of freedom and the standard error of the mean participant-level proportion. This approach accounted for the grouping of multiple responses within participants rather than treating all item responses as independent observations. The expected accuracy under uniform random selection was calculated as a descriptive benchmark; no hypothesis test against this benchmark was performed.

Subgroup comparisons of total scores according to professional status, professional experience, and institution type used the Kruskal–Wallis test with correction for tied ranks. The corresponding effect size was calculated as max[0, (H − k + 1)/(N − k)], where H is the test statistic, k is the number of groups, and N is the total sample size. Scores of participants with and without previous healthcare-cost education were compared using a two-sided Mann–Whitney U test with a normal approximation incorporating tie and continuity corrections. Rank-biserial correlation was calculated as 1 − 2U/(n_1_n_2_), with U defined for the previously educated group. No post hoc pairwise comparisons were performed.

An ordinary least-squares multivariable linear regression model examined associations between participant characteristics and the total tariff-response score. Predictors were approximate age, professional-status group, institution-type group, and previous healthcare-cost education. Professional status was grouped as resident, specialist, or academic physician, with residents as the reference category. Academic physicians included assistant professors, associate professors, and professors. Secondary- and tertiary-level public hospitals were combined into a public-hospital reference category; the remaining categories were university hospital, private hospital, and other. Participants without previous healthcare-cost education formed the reference group for that predictor.

Age was collected categorically and assigned approximate values of 27.0, 35.5, 45.5, 55.5, and 65.0 years for the categories 24–30, 31–40, 41–50, 51–60, and >60 years, respectively. The derived variable was divided by 10 so that its coefficient represented a 10-year increase in assigned age. The value for the open-ended oldest category was an analytical approximation. Sex was not included because only three participants were female.

The regression model included an intercept and seven predictor parameters. HC3 heteroscedasticity-robust standard errors were used for coefficient inference. Unstandardized coefficients were reported with 95% confidence intervals and two-sided *p*-values based on the t distribution with 94 residual degrees of freedom. Overall model significance was evaluated using an HC3 robust Wald F test of the joint null hypothesis that all seven predictor coefficients were zero. Model fit was summarized using R^2^ and adjusted R^2^, and Cohen’s f^2^ was calculated as R^2^/(1 − R^2^).

Hypothetical scenario responses were summarized separately for each question as frequencies and percentages, using 102 participants as the denominator. The free-text response to the initial-approach question in Scenario 1 was retained in the “Other” category. Zero-frequency response options were also reported. Because the questions used non-equivalent response formats and some response combinations were internally inconsistent, no paired analysis or combined indicator of decision change was calculated.

All inferential tests were two-sided, with *p* < 0.05 used as the nominal significance threshold. No adjustment for multiple comparisons was applied. Subgroup comparisons and multivariable regression analyses were secondary and exploratory. They examined associations with the unweighted total number of reference-matching responses, rather than determinants of a validated knowledge construct. Findings were interpreted in light of heterogeneous response formats, the absence of adjustment for guessing, multiple testing, sparse categories, and the limited sample size. Nonsignificant findings were not interpreted as evidence of equivalence or absence of an association.

## 3. Results

### 3.1. Participant Characteristics

A total of 102 complete questionnaires were included in the analysis. Participants were predominantly male (*n* = 99, 97.1%). The largest age group was 31–40 years (*n* = 44, 43.1%), followed by 41–50 years (*n* = 22, 21.6%) and 24–30 years (*n* = 18, 17.6%). The sample comprised 27 residents (26.5%), 38 specialists (37.3%), 8 assistant professors (7.8%), 16 associate professors (15.7%), and 13 professors (12.7%).

Forty-eight participants (47.1%) worked in tertiary public hospitals, 19 (18.6%) in university hospitals, 15 (14.7%) in private hospitals, 13 (12.7%) in secondary public hospitals, and 7 (6.9%) in other institutions. Six participants (5.9%) reported previous education concerning healthcare costs. Participant characteristics are presented in Table 1.

### 3.2. Accuracy of Reimbursement Responses

The mean number of reference-matching responses across the 18 items was 6.02 ± 2.38, with a median of 6 (IQR, 4–8) and a range of 2–12. Overall, 614 of 1836 responses matched the scoring key, corresponding to a mean participant-level accuracy of 33.4% (95% CI, 30.8–36.0%). The theoretical expected accuracy under uniform random selection among the available options was 31.5%.

Observed correct-response proportions were 38.9% (95% CI, 34.0–43.8%) for plain radiography, 40.2% (95% CI, 32.4–48.0%) for computed tomography, 25.5% (95% CI, 17.7–33.3%) for magnetic resonance imaging, 22.1% (95% CI, 18.0–26.1%) for outpatient procedures, 22.2% (95% CI, 17.0–27.5%) for surgical procedures, and 52.9% (95% CI, 46.7–59.1%) for implants.

At the item level, the highest correct-response proportion was observed for single-view pelvis radiography (66/102, 64.7%), followed by cortical screws (59/102, 57.8%), proximal humerus plates (57/102, 55.9%), and knee CT (54/102, 52.9%). The lowest proportions were observed for trigger-finger surgery (8/102, 7.8%), primary partial large-joint prosthesis (13/102, 12.7%), closed reduction of a major bone fracture (14/102, 13.7%), and shoulder MRI (21/102, 20.6%). Complete item-level and category-level results are presented in Table 2.

### 3.3. Subgroup Comparisons

In the secondary, exploratory subgroup analyses, no statistically significant differences in total tariff-response scores were detected according to professional status, professional experience, institution type, or previous healthcare-cost education (all *p* > 0.05). Group-specific descriptive statistics, test results, and effect-size estimates are provided in Supplementary Table S1.

### 3.4. Multivariable Analysis

The secondary, exploratory multivariable linear regression model explained a small proportion of the observed variance in total tariff-response scores (R^2^ = 0.063; adjusted R^2^ = −0.007). The HC3 robust overall Wald test was not statistically significant (F[7,94] = 0.99, *p* = 0.447), and no statistically significant associations were detected between the included predictors and the total score. Full coefficient estimates, HC3 heteroscedasticity-robust standard errors, 95% confidence intervals, and *p*-values are presented in Supplementary Table S2.

### 3.5. Responses to Hypothetical Clinical Scenarios

In Scenario 1, medical treatment plus exercise or physical therapy was the most frequently selected initial approach (72/102, 70.6%). In Scenario 2, 99 participants (97.1%) indicated that they would request advanced imaging after radiography, with CT being the most frequently selected first imaging choice (85/102, 83.3%). In Scenario 3, 89 participants (87.3%) indicated that they would request ankle radiography. CT was selected by 16 participants (15.7%) when the radiograph was normal and by 98 (96.1%) in the alternative case involving surgical planning.

Responses to the conditional questions about hypothetical disclosure of SUT prices are presented alongside the complete distributions for each scenario question in Table 3. All responses were summarized descriptively. The non-equivalent response formats and internally inconsistent response combinations precluded a reliable assessment of participant-level decision change.

## 4. Discussion

Overall, 614 of 1836 responses matched the reference-based scoring key (33.4%), and the mean total tariff-response score was 6.02 ± 2.38 out of 18. The theoretical expected accuracy under uniform random selection was 31.5%. The numerical proximity of these values warrants caution when interpreting the observed accuracy, but it does not establish that participants guessed. No formal comparison with this benchmark was performed.

Category-level accuracy ranged from 22.1% for outpatient procedures to 52.9% for implants. Differences in the number of response options and the widths of monetary intervals limit comparisons between categories. The secondary, exploratory subgroup and regression analyses identified no statistically significant differences or associations. The regression model explained little of the observed score variance (R^2^ = 0.063; adjusted R^2^ = −0.007), and the modest sample size and small subgroup sizes limited the precision of these estimates.

### 4.1. Comparison with Previous Studies

The overall accuracy of 33.4% was numerically similar to the 33% reported in the systematic review by Allan and Lexchin [1]. However, the reviewed studies examined different monetary concepts and generally defined accuracy as an estimate within 20% or 25% of a reference value. The present study used multiple-choice amounts or intervals. Similar percentages obtained with different instruments and scoring criteria do not indicate equivalent levels of knowledge.

In orthopedic practice, Okike et al. found that attending surgeons estimated implant acquisition costs within 20% of the reference value in 21% of responses, compared with 17% among residents [4]. Schultz et al. reported that 19.4% of estimates for trauma-related imaging were within 20% of institutional charges, with no statistically significant difference between residents and faculty members [6]. In the present study, accuracy was 38.9% for radiography, 40.2% for CT, 25.5% for MRI, and 52.9% for implants. These numerically higher proportions in some categories should be interpreted in light of differences in monetary concepts and response formats. Selecting a supplied reimbursement interval differs from producing an open numerical estimate and allows correct responses through guessing.

In Türkiye, Sarbay et al. reported a mean accuracy of 17.7% among 270 emergency medicine residents estimating monetary values for commonly ordered diagnostic tests. Only 2.6% reported relevant education, and no statistically significant differences in accuracy were detected by hospital type or professional experience [7]. In the present sample, 5.9% reported previous healthcare-cost education, and comparisons by institution type and experience were also nonsignificant. Although differences in specialty, services, reference values, and assessment methods prevent direct comparison of accuracy, both samples included few participants with previous relevant education.

Financial information may also be relevant to patient communication. Au et al. reported limited self-perceived awareness of metallic implants among orthopedic patients in Singapore, with 51% identifying surgical cost as an important concern [11]. Although that study did not assess physicians’ reimbursement knowledge, it suggests that financial questions may arise during preoperative discussions alongside clinical effectiveness, implant characteristics, risks, alternatives, and patient preferences.

Implant questions had the highest observed accuracy in the present study. Nevertheless, clinical involvement in implant selection does not necessarily imply familiarity with associated monetary values. Chan et al. reported limited knowledge of implant costs and healthcare financing schemes among orthopedic residents, while Rohman et al. reported limited implant-cost knowledge among consultant orthopedic surgeons [12,13]. These findings provide context for the present results, although differences in instruments and payment systems restrict direct comparisons.

The present study contributes a descriptive account of responses across several areas of orthopedic practice within the Turkish reimbursement context. Its findings are specific to the selected questions and participating physicians.

### 4.2. Educational Implications

Six participants (5.9%) reported previous healthcare-cost education. Sinkler et al. also described limited familiarity with healthcare costs among orthopedic residents and discussed the potential role of residency education [14]. The nonsignificant comparison by previous education in the present study cannot establish whether such education is effective. Only six participants reported previous education, and its timing, duration, content, and quality were not recorded.

Educational content should clearly distinguish administrative reimbursement amounts from institutional costs, acquisition prices, patient payments, and societal costs. For example, Fidanza et al. used time-driven activity-based costing to examine inpatient costs for total hip arthroplasty and identified the prosthetic device and hospitalization as major components [15]. This resource-based approach measures a different quantity from an SUT tariff. Understanding these distinctions may be a useful educational objective alongside familiarity with reimbursement systems.

Educational interventions should also be evaluated for their clinical consequences. Hirani et al. discussed potential benefits and challenges of strategies intended to reduce hospital length of stay [16], illustrating the need to consider efficiency together with patient safety and clinical effectiveness. Whether tariff education improves decisions, reduces resource use, or produces savings requires prospective evaluation.

### 4.3. Implant Information, Transparency, and Professional Safeguards

Reliable implant-related financial information may support educational efforts. Streit et al. reported substantial errors in surgeons’ estimates of implant costs [17]. The United States Government Accountability Office also documented variation in hospital payments for implantable devices and described how confidentiality provisions could restrict price transparency [18]. These findings support further investigation of how financial information is communicated, including clear identification of whether displayed values represent acquisition prices, charges, or reimbursement amounts. The present study did not evaluate which format would be most useful.

Educational resources should also address relationships between orthopedic surgeons and device manufacturers. Previous investigations highlight the importance of transparency and conflict-of-interest oversight [19]. Institutional or professional-society involvement, disclosure of financial relationships, and clear identification of manufacturers’ roles are relevant considerations when developing such resources.

Stammen et al. identified knowledge transmission, reflective practice with feedback, and a supportive clinical environment as components associated with education in high-value, cost-conscious care [20]. Yip et al. used online pediatric musculoskeletal cases to examine learners’ diagnostic choices in relation to monetary information [21]. These approaches offer possible models for future orthopedic education, but their effectiveness in Türkiye would require direct evaluation using clearly defined financial information and appropriate clinical outcomes.

### 4.4. Interpretation of the Hypothetical Scenarios

The scenario findings describe stated preferences in response to individual hypothetical questions, including conditional questions about disclosure of SUT prices. They do not represent a standardized tariff-display intervention. Non-equivalent response options and internally inconsistent response combinations prevented reliable assessment of participant-level decision change. Reporting each question separately therefore preserves the descriptive information without attributing changes in behavior to reimbursement information.

Roedbro and Jervelund reviewed European studies of laboratory-test price display and reported associations with reduced ordering or expenditure, while noting limitations in the assessment of sustained effects and patient safety [22]. This intervention literature addresses a different question from the present survey. Future evaluations should assess clinical indications, diagnostic consequences, patient outcomes, and expenditure together. They should also examine potential unintended effects, such as omission of indicated investigations or unequal application of financial considerations.

### 4.5. Limitations

This study has several limitations. First, recruitment through the TOTBİD e-mail group was voluntary and used a nonprobability sampling approach. The number and characteristics of nonrespondents were unavailable, preventing calculation of a response rate or assessment of nonresponse bias. Geographic locations were not recorded, so regional coverage could not be verified. National dissemination of the invitation does not establish national representativeness, and the findings cannot be generalized to all orthopedic and trauma physicians in Türkiye.

Second, no a priori sample-size calculation was performed. The final sample comprised 102 participants and was predominantly male. Small subgroup sizes, particularly the six participants reporting previous healthcare-cost education, limited the precision of the secondary, exploratory analyses. No adjustment for multiple comparisons was applied, and nonsignificant findings should not be interpreted as evidence of equivalence or absence of an association. Age was approximated from categories, including an assigned value for the open-ended oldest category, so the age coefficient does not reflect exact participant ages.

Third, the study-specific questionnaire did not undergo formal pilot testing, external expert-panel review, or psychometric validation. The 18 reimbursement questions varied in their number of options and monetary interval widths, with some options specifying individual amounts. The unweighted score was not adjusted for guessing or differences in item difficulty and should be interpreted as a descriptive count of reference-matching responses rather than a validated measure of comprehensive health-economic knowledge.

Fourth, the questionnaire used the term “cost” while instructing participants to refer to SUT amounts, which may have affected interpretation of the questions. The findings concern selected administrative reimbursement amounts, and subsequent changes to tariffs may limit their applicability over time.

Fifth, the clinical scenarios were hypothetical and self-reported, making them susceptible to social-desirability bias. Their measurement limitations are described in Section 4.4. No actual ordering behavior, clinical appropriateness, patient outcomes, access inequalities, or expenditure was assessed.

Finally, the cross-sectional design precludes causal inference. Future studies should use validated instruments, clearly defined monetary concepts, equivalent measurements when assessing change, and prospectively specified clinical and economic outcomes.

### 4.6. Strengths

The questionnaire covered imaging examinations, outpatient procedures, surgical interventions, and implants within a single study. Participation by physicians at different professional levels and from different institution types enabled exploratory comparisons within the sample. The range of topics covered may help guide item selection for future standardized assessments.

## 5. Conclusions

In this voluntary sample of 102 orthopedic and trauma physicians in Türkiye, 33.4% of responses matched the reference-based scoring key for selected SUT reimbursement amounts. Interpretation is limited by differences in response options and interval widths, the possibility of correct responses through guessing, and the nonrepresentative sample. The findings concern selected administrative reimbursement amounts and do not measure actual healthcare costs or comprehensive health-economic competence.

The hypothetical scenario responses are descriptive and do not establish individual decision changes or effects of tariff information on clinical practice, appropriateness, utilization, expenditure, or patient outcomes. Future studies should use validated instruments, standardized response formats, and more representative samples. The effectiveness and safety of reimbursement education or financial-information interventions require prospective evaluation.

## Figures and Tables

**Table 1:** Participant characteristics (*N* = 102).

Variable	Category	*n* (%)
Age group (years)	24–30	18 (17.6)
	31–40	44 (43.1)
	41–50	22 (21.6)
	51–60	16 (15.7)
	>60	2 (2.0)
Sex	Male	99 (97.1)
	Female	3 (2.9)
Professional status	Resident	27 (26.5)
	Specialist	38 (37.3)
	Assistant professor	8 (7.8)
	Associate professor	16 (15.7)
	Professor	13 (12.7)
Institution	Secondary public hospital	13 (12.7)
	Tertiary public hospital	48 (47.1)
	University hospital	19 (18.6)
	Private hospital	15 (14.7)
	Other	7 (6.9)
Professional experience	0–2 years	8 (7.8)
	3–5 years	18 (17.6)
	6–10 years	26 (25.5)
	11–15 years	22 (21.6)
	16–20 years	10 (9.8)
	>20 years	18 (17.6)
Monthly outpatient clinic days	0–10	46 (45.1)
	11–20	39 (38.2)
	21–31	17 (16.7)
Monthly surgical procedures	0–10	25 (24.5)
	11–20	46 (45.1)
	21–30	16 (15.7)
	>30	15 (14.7)
Previous healthcare-cost education	No	96 (94.1)
	Yes	6 (5.9)

**Table 2:** Accuracy of reimbursement responses.

Category/Item	Correct, *n*/*N*	Accuracy (%)	95% CI (%)
Overall	614/1836	33.4	30.8–36.0
Plain radiography	119/306	38.9	34.0–43.8
Two-view right knee radiograph	23/102	22.5	15.5–31.6
Single-view pelvis radiograph	66/102	64.7	55.1–73.3
Lower-limb length radiograph	30/102	29.4	21.4–38.9
Computed tomography	123/306	40.2	32.4–48.0
Right knee CT	54/102	52.9	43.3–62.3
Pelvis CT	45/102	44.1	34.9–53.8
Lumbar spine CT	24/102	23.5	16.4–32.6
Magnetic resonance imaging	52/204	25.5	17.7–33.3
Right knee MRI	31/102	30.4	22.3–39.9
Left shoulder MRI	21/102	20.6	13.9–29.4
Outpatient procedures	90/408	22.1	18.0–26.1
Short-arm cast	45/102	44.1	34.9–53.8
Intra-articular injection	23/102	22.5	15.5–31.6
Trigger-finger surgery	8/102	7.8	4.0–14.7
Closed reduction of a major bone fracture	14/102	13.7	8.4–21.7
Surgical procedures	68/306	22.2	17.0–27.5
Primary partial large-joint prosthesis	13/102	12.7	7.6–20.6
Total knee arthroplasty	24/102	23.5	16.4–32.6
Arthroscopic ACL reconstruction	31/102	30.4	22.3–39.9
Implants	162/306	52.9	46.7–59.1
Cortical screw	59/102	57.8	48.1–67.0
Proximal humerus plate	57/102	55.9	46.2–65.1
Acetabular cup	46/102	45.1	35.8–54.8

Overall and category confidence intervals are Student t intervals for mean participant-level correct-response proportions (*N* = 102). Item-level intervals are Wilson intervals. The same reference-based key was applied to all participants. CT, computed tomography; MRI, magnetic resonance imaging; ACL, anterior cruciate ligament.

**Table 3:** Responses to hypothetical clinical scenarios.

Scenario/Question	Response Option	*n* (%)
Q32. Scenario 1: Initial approach	Order MRI	8 (7.8)
	Medical treatment plus exercise/physical therapy	72 (70.6)
	Follow-up clinical examination	21 (20.6)
	Emergency orthopedic assessment/further evaluation	0 (0.0)
	Other (free-text response)	1 (1.0)
Q33. Scenario 1: MRI at first visit	Yes	8 (7.8)
	No	89 (87.3)
	Undecided	5 (4.9)
Q34. Scenario 1: Would your decision change if the SUT price of knee MRI were shown?	Yes, I would not request MRI	7 (6.9)
	No, I would still request MRI	14 (13.7)
	I would not have requested MRI anyway	75 (73.5)
	Undecided	6 (5.9)
Q35. Scenario 2: Advanced imaging after radiography	Yes	99 (97.1)
	No	2 (2.0)
	Undecided	1 (1.0)
Q36. Scenario 2: First imaging choice	Hip/pelvis CT	85 (83.3)
	Hip/pelvis MRI	14 (13.7)
	Follow-up radiograph	2 (2.0)
	Scintigraphy	0 (0.0)
	I would not request advanced imaging	1 (1.0)
Q37. Scenario 2: Approach with high suspicion of occult hip fracture	CT	66 (64.7)
	MRI	33 (32.4)
	Admission and observation plus follow-up radiography	2 (2.0)
	Discharge plus outpatient follow-up	1 (1.0)
	Other	0 (0.0)
Q38. Scenario 2: Would your preference change if SUT prices were shown?	Yes, I would prefer CT	15 (14.7)
	Yes, I would prefer MRI	3 (2.9)
	No, I would request the examination I consider clinically necessary	84 (82.4)
	I would not request advanced imaging	0 (0.0)
	Undecided	0 (0.0)
Q39. Scenario 3: Ankle radiography	Yes	89 (87.3)
	No	9 (8.8)
	Undecided	4 (3.9)
Q40. Scenario 3: CT if radiograph normal	Yes	16 (15.7)
	No	80 (78.4)
	Undecided	6 (5.9)
Q41. Scenario 3: CT for surgical planning in the alternative case	Yes	98 (96.1)
	No	3 (2.9)
	Undecided	1 (1.0)
Q42. Scenario 3: Would your decision change if SUT prices were shown?	Yes, I would not request radiography	3 (2.9)
	Yes, I would not request CT	1 (1.0)
	No, I would request it if clinically indicated	97 (95.1)
	I would not have requested CT anyway	0 (0.0)
	Undecided	1 (1.0)

*N* = 102 for each question. Question numbers refer to the original questionnaire. Questions are summarized separately, not as paired before-and-after observations. Percentages may not sum to 100 because of rounding.

## Data Availability

The data supporting the findings of this study are available from the corresponding author upon reasonable request.

## Supplementary Materials

The following supporting information can be downloaded at https://www.mdpi.com/article/10.3390/healthcare14193284/s1. Supplementary Table S1. Exploratory subgroup comparisons, Supplementary Table S2. Exploratory multivariable linear regression.

## Abbreviations

The following abbreviations are used in this manuscript:

ACL	Anterior Cruciate Ligament
CI	Confidence Interval
CT	Computed Tomography
HC3	Heteroscedasticity-Consistent Standard Error Type 3
IQR	Interquartile Range
MRI	Magnetic Resonance Imaging
SD	Standard Deviation
SGK	Social Security Institution of the Republic of Türkiye
SUT	Health Implementation Communiqué (Sağlık Uygulama Tebliği)
TDABC	Time-Driven Activity-Based Costing
TL	Turkish Lira
TOTBİD	Turkish Society of Orthopedics and Traumatology
