# Supplementary material for: Orthopedic and Trauma Physicians’ Ability to Identify Selected SUT Reimbursement Amounts in Türkiye: A Cross-Sectional Survey
Source: Healthcare (Basel). 2026 Oct 2;14(19):3284. doi: 10.3390/healthcare14193284 (PMC13665003; doi:10.3390/healthcare14193284)
Supplement: Supplementary file 1 [file healthcare-14-03284-s001.zip › healthcare-4576489-supplementary.pdf]

Supplementary Table S1. Exploratory subgroup comparisons

Variable	Category	n	Median (IQR)	H / U	p	Effect size
Professional status	Resident	27	6.0 (4.0–6.0)	5.69	0.224	0.017
	Specialist	38	7.0 (4.0–8.0)			
	Assistant professor	8	4.5 (3.0–6.2)			
	Associate professor	16	6.5 (4.8–9.0)			
	Professor	13	7.0 (4.0–8.0)			
Professional experience	0–2 years	8	5.0 (4.8–6.0)	2.27	0.811	0.000
	3–5 years	18	6.0 (4.2–7.0)			
	6–10 years	26	6.5 (4.0–8.0)			
	11–15 years	22	5.5 (4.0–8.0)			
	16–20 years	10	7.0 (4.2–9.0)			
	>20 years	18	6.0 (4.0–8.0)			
Institution	Secondary public hospital	13	5.0 (4.0–7.0)	4.48	0.345	0.005
	Tertiary public hospital	48	6.0 (4.0–7.0)			
	University hospital	19	6.0 (4.0–8.0)			
	Private hospital	15	8.0 (5.5–9.0)			
	Other	7	6.0 (4.0–9.0)			
Previous healthcare-cost education	No	96	6.0 (4.0–8.0)	268.00	0.780	0.069
	Yes	6	5.0 (5.0–5.8)			

H: tie-corrected Kruskal–Wallis statistic; U: Mann–Whitney statistic for previous education (yes versus no), using a two-sided normal approximation with tie and continuity correction. Kruskal–Wallis effect size = max[0, (H − k + 1)/(N − k)], where k is the number of groups. Rank-biserial correlation for education = 1 − 2U/(6 × 96); its sign follows this stated convention. IQRs use linear interpolation.

Supplementary Table S2. Exploratory multivariable linear regression

**Predictor**	**B**	**HC3 SE**	**95% CI**	**t**	**p**
Approximate age (per 10-year increase)	0.02	0.45	-0.87 to 0.92	0.05	0.958
Specialist versus resident	0.73	0.71	-0.67 to 2.13	1.03	0.305
Academic versus resident	0.26	0.90	-1.52 to 2.04	0.29	0.770
University versus public hospital	0.51	0.83	-1.15 to 2.17	0.61	0.543
Private versus public hospital	1.36	0.84	-0.31 to 3.04	1.62	0.109
Other versus public hospital	0.76	1.25	-1.73 to 3.24	0.60	0.547
Previous education: yes versus no	-0.53	0.89	-2.30 to 1.23	-0.60	0.551

N = 102. $R^2 = 0.063$; adjusted $R^2 = -0.007$; Cohen's $f^2 = 0.067$. HC3 robust overall Wald $F(7, 94) = 0.99$, $p = 0.447$. Coefficient confidence intervals and p-values use HC3 standard errors and a t distribution with 94 residual degrees of freedom. B denotes the unstandardized coefficient.

Age categories were assigned values of 27.0, 35.5, 45.5, 55.5, and 65.0 years for ages 24–30, 31–40, 41–50, 51–60, and >60, respectively. These are analytical approximations, not exact ages. Academic physicians include assistant professors, associate professors, and professors. Public hospitals include secondary- and tertiary-level public hospitals. The model included an intercept.
